# Association of *Encephalitozoon cuniculi* with Clinical Signs and Abnormal Hematologic/Biochemical Changes in Pet Rabbits in Thailand

**DOI:** 10.3390/ani14192766

**Published:** 2024-09-25

**Authors:** Taksaon Duangurai, Natruree Khamchomphu, Kanyanut Dusitkul, Chawaporn Tousee, Yosanun Sukmai, Teerapat Rungnirundorn, Ladawan Areevijittrakul, Siriluk Jala, Naris Thengchaisri

**Affiliations:** 1 Department of Companion Animal Clinical Sciences, Faculty of Veterinary Medicine, Kasetsart University, Bangkok 10900, Thailand; taksaon.du@ku.th; 2 Kasetsart University Veterinary Teaching Hospital, Faculty of Veterinary Medicine, Kasetsart University, Bangkok 10900, Thailand; natruree.k@ku.th (N.K.); kam-beast@hotmail.co.th (K.D.); kinko_jang@hotmail.com (C.T.); chain_23@hotmail.co.th (Y.S.); estel1819@hotmail.com (T.R.); ladawan.a@ku.th (L.A.); 3 Kamphaeng Saen Veterinary Diagnosis Center, Faculty of Veterinary Medicine, Kasetsart University Kamphaeng Saen Campus, Nakhon Pathom 73140, Thailand; siriluk.j@ku.th

**Keywords:** anemia, antibody status, encephalitozoonosis, pet rabbits, subclinical infection

## Abstract

**Simple Summary:**

This study explores the impact of the *Encephalitozoon cuniculi* on pet rabbits. It examines the link between antibody levels against this parasite and various health parameters in pet rabbits. By dividing 90 pet rabbits into healthy, subclinical, and clinical groups, this study carefully analyzed blood and biochemical changes. Key findings reveal that higher antibody levels in subclinical and clinical rabbits are crucial for diagnosis and management. This study also associates neurological signs and anemia with active infection, emphasizing the need for vigilant clinical and hematologic monitoring. The research found a higher infection rate in mature adult rabbits and a significant prevalence in Bangkok, indicating regional and age-related risk factors. Anemia was significantly linked to *E. cuniculi* infection, while no connections were found with other parameters. Recognizing anemia and neurological symptoms aids early diagnosis.

**Abstract:**

*Encephalitozoon cuniculi* can cause serious disease and subclinical infection in rabbits and requires active surveillance to control the infection. This study investigated the association between anti-*Encephalitozoon cuniculi* antibody status and various health parameters in pet rabbits. A total of 90 rabbits were divided into healthy (*N* = 30), subclinical (*N* = 30), and clinical (*N* = 30) groups based on their anti-*Encephalitozoon cuniculi* antibody status and clinical presentations. The mean ages of the control (37 ± 40 months) and subclinical groups (38 ± 34 months) were notably lower compared to that of the clinical group (63 ± 38 months, *p* < 0.01). Serum titers for anti-*Encephalitozoon cuniculi* antibodies were significantly elevated in rabbits with subclinical and clinical infections compared to those of healthy rabbits (*p* < 0.05). Neurological signs were predominant in rabbits with active *E. cuniculi* infection (80.0%), with additional pathological features including urinary dysfunction (10.0%) and cataracts (10.0%). The source of rabbits was not associated with *E. cuniculi* infection (*p* = 0.159). Anemia was significantly linked to *E. cuniculi* infection (*p* = 0.026); however, no significant associations were found with leukocytosis, thrombocytopenia, or serum biochemistry parameters. Mature adult rabbits were more likely to be infected with *E. cuniculi*. Recognizing anemia and neurological signs facilitates in early diagnosis of *E. cuniculi* infection.

## 1. Introduction

*Encephalitozoon cuniculi* is an obligate intracellular and spore-forming microsporidian parasite. This parasite can infect various mammalian hosts, such as rabbits, rodents, dogs, cats, horses, ruminants, non-human primates, and humans [1]. Hosts with compromised immune systems are especially vulnerable to *E. cuniculi* infection [1,2]. Rabbits and other animals, including non-human primates, can serve as a source of *E. cuniculi* infection, posing a possible zoonotic risk [2,3]. It is important to be attentive to the zoonotic potential of the *E. cuniculi* pathogen.

*E. cuniculi* typically enters the gastrointestinal or respiratory epithelium through the extrusion of its polar filament and injection of infectious sporoplasm (containing genetic machinery and DNA) into the host cell [1,2]. Upon contact with a host cell, changes in the osmotic pressure and pH trigger the extrusion of the polar filament. This allows the sporoplasm to enter the new host cell, in which it develops into a meront. The meront then undergoes a process called merogony, during which it divides to form sporonts [1,2]. Although cellular phagocytosis of the spore by host cells has been observed, it is considered a less significant route of infection. The final stage of *E. cuniculi* development occurs within a parasitophorous vacuole that forms inside the host cell’s cytoplasm. Inside this vacuole, sporonts mature into sporoblasts, which produce the polar tube and other components necessary to form mature spores [1,2]. When the parasitophorous vacuole ruptures, the mature, infectious spores are released into the bloodstream [1,2].

*E. cuniculi* can be transmitted both horizontally and vertically (transplacentally). In rabbits, horizontal transmission commonly occurs through the ingestion of urine-contaminated feed (affecting the small intestine) or through the inhalation of spores (affecting the respiratory tract) [2]. After ingestion or inhalation, the spores penetrate the epithelial cells and replicate through the reticuloendothelial system, distributing the parasite to other organs, including the liver, kidneys, central nervous system (CNS), lungs, and heart, via infected macrophages. This spread often leads to inflammatory lesions due to the rupture of infected host cells and the release of spores in different organs [2]. Transplacental transmission can result in intraocular infection in young rabbits, causing uveitis [4]. Ocular lesions associated with this infection typically present as phacoclastic uveitis, characterized by a yellow-white granuloma, and can lead to cataracts [1,5,6].

The clinical signs of encephalitozoonosis depend on the rabbit’s immune status. Immunosuppressed rabbits show severe clinical signs that often lead to the death of the animal [2,7,8]. Clinical signs of *E. cuniculi* infection in rabbits include neurological issues such as seizures, paresis, head tilt, ataxia, and circling. Indicators of renal failure like azotemia, polyuria, polydipsia, and pollakiuria are also observed [2]. A significant portion of infected individuals remain asymptomatic but may suffer from unreported health issues. The accurate detection of *E. cuniculi* infection is crucial for proper treatment and requires [1,9] specialized staining or electron microscopy in laboratory diagnostics. A serological test is one of the most widely used techniques to detect *E. cuniculi* antibody titer in live rabbits [3,10,11,12]. However, the utility of this method is quite limited. A positive result only indicates that the animals have previous exposure to *E. cuniculi*, without providing information as to when the infection occurred [13]. Clinical signs are not necessarily associated with a high antibody titer [13]. A negative result for antibody titer can rule out encephalitozoonosis [1,13]. However, DNA-based methods used to detect *E. cuniculi* from feces, urine, and cerebrospinal fluid samples have been reported to exhibit low sensitivity. The potential for false negative results may suggest higher rates of *E. cuniculi* infection than those reported [14]. The existence of subclinical *E. cuniculi* infection also underscores the potential widespread prevalence of the disease among both pet and meat rabbits. In Thailand, *E. cuniculi* seroprevalence was observed in meat rabbits ranging from 42.4% to 70.4%, while pet rabbits displayed a seroprevalence of 67.1% [15]. Rabbits hold economic significance for protein production in many regions, and a high seroprevalence of *E. cuniculi* in rabbit farms can negatively impact this industry.

The connection between *E. cuniculi* seroprevalence and hematologic parameters has not been well-characterized. However, hematological markers may be useful in predicting disease in asymptomatic animals. The objectives of the present study were to compare hematological changes, including anemia, leukocytosis, and thrombocytopenia, as well as biochemical alterations among the rabbits classified as clinically, subclinically infected, and non-infected. The potential association between infection status and various factors, including rabbit origin, province distribution, and abnormal clinical parameters, was also determined across the *E. cuniculi* infective status. A major goal of this analysis is to mitigate the zoonotic risk for individuals involved in animal-assisted interventions (AAIs).

## 2. Materials and Methods

### 2.1. Study Population

All animal procedures were approved by the Kasetsart University Institutional Animal Care and Use Committee (ACKU64-VET-023, License No. U1-00723-2558). Prior to sample collection, owners provided informed consent by signing consent forms. A total of 90 pet rabbit patients visiting Kasetsart University Veterinary Teaching Hospital between 2020 and 2022 were enrolled in the *E. cuniculi* survey. Clinical histories were taken, including, age, gender, neuter history, husbandry, and health status. This study included 39 male and 51 female rabbits with a median body weight of 1.4 kg (range: 0.8–5.1 kg) and a median age of 3.0 years (range: 0.4–11.8 years). The breeds represented were 30 Holland Lops, 14 Netherland Dwarves, 2 Flemish Giants, 1 Rex, and 43 mixed breeds. External physical examinations were performed for each rabbit. Neurological signs were evaluated, including head tilt, ataxia, paralysis, circling, rolling, nystagmus, and tremors, as well as clinical signs of renal disease, including urinary incontinence, polyuria, polydipsia, and analytical abnormality, including azotemia, and ophthalmic abnormalities, including cataracts, uveitis, and glaucoma. The 90 rabbits were classified into the following three groups depending on the stages of infection: Group 1 (healthy) included rabbits negative for anti-*E. cuniculi* antibodies according to an ELISA (*N* = 30); Group 2 (subclinical) included apparently healthy rabbits that tested positive for anti-*E. cuniculi* antibodies but showed no clinical signs of encephalitozoonosis (no neurological, urinary, ocular, or gastrointestinal signs; *N* = 30). All rabbits in Group 3 (clinical) were positive for anti-*E. cuniculi* antibodies according to an ELISA and exhibited one or more clinical signs associated with encephalitozoonosis, such as vestibular disease (head tilt, nystagmus, circling, rolling, or imbalance), urinary system issues (polyuria, polydipsia, dehydration, anorexia, weight loss, sludgy urine, urine scalding, and difficulty urinating), eye disorders (cataract, uveitis, or eye granuloma), and gastrointestinal signs (gastrointestinal hypomotility, cachexia, anorexia, and chronic weight loss; *N* = 30) (Table 1).

### 2.2. Hematological and Biochemical Analysis

Approximately 1 mL of blood was collected from the saphenous vein. 0.5 mL of ethylenediamine tetra-acetic acid (EDTA) and 0.5 mL of heparinized blood samples were sent to the hematology unit at the Kasetsart University Veterinary Teaching Hospital, Faculty of Veterinary Medicine (Bangkok, Thailand) for hematological and biochemical analysis. The EDTA blood samples were transferred at ambient temperature for hematological analysis using the Sysmex XN-1000V hematology analyzer (Sysmex, Kobe, Japan). A complete blood count (CBC) was performed to assess various parameters, including packed cell volume (PCV), white blood cell count (WBC), mean corpuscular hemoglobin concentration, mean corpuscular volume, white blood cell count, neutrophil count, lymphocyte count, monocyte count, eosinophil count, and platelet count (PLT), in order to detect anemia and leukocytosis. Additionally, heparinized blood samples were collected for biochemical tests. Blood urea nitrogen (BUN), creatinine (CREA), aspartate aminotransferase (AST), alkaline phosphatase (ALP), total protein, and glucose level tests were performed using the VetScan VS2 Chemistry Analyzer (Abaxis, Union City, CA, USA).

### 2.3. Preparation of Rabbit Sera

Blood collected from the saphenous vein was allowed to clot by leaving the collection tube undisturbed for 30 min at room temperature. Sera were then harvested using centrifugation at 2000 relative centrifugal force (RCF) for 10 min at 4 °C, and samples were stored at −20 °C until use. Three biological replicates were obtained. Rabbit sera were tested for antibodies to *E. cuniculi* with ELISA.

### 2.4. Detection of Antibodies against E. cuniculi

To detect antibodies against *E. cuniculi*, we employed an *E. cuniculi* IgG ELISA test kit from XpressBio (Frederick, MD, USA). The ELISA plate included paired wells containing positive and negative control antigens, which serve as reference points to ensure the accuracy of the assay. Serum samples were diluted in a series to create a range of concentrations, allowing for the detection of varying levels of antibodies. Horseradish peroxidase-conjugated anti-rabbit IgG was used as the secondary antibody, which binds specifically to the rabbit IgG present in the samples. ABTS (2,2′-azino-bis (3-ethylbenzothiazoline-6-sulfonic acid)) was used as the substrate, which reacts with the enzyme conjugate to produce a measurable color change, indicating the presence and quantity of *E. cuniculi* antibodies in the serum. Absorbance was measured at a wavelength of 405 nm using an ELISA reader (Tecan, Männedorf, Switzerland) to quantify the color change resulting from the enzymatic reaction. To determine the IgG antibody titer, the absorbance value of each sample was divided by the absorbance of the positive control antigen, creating an index of reactivity. Samples with an index value of 0.3 or higher were classified as positive for anti-*E. cuniculi* antibodies, indicating a significant presence of the antibody in the serum. Notably, the ELISA kit used is a standardized and validated procedure, recognized for its reliability. Despite the 3-year sample collection period, its consistency ensures accurate and comparable results across all time points.

### 2.5. Statistical Analysis

Individual characteristics of rabbits, such as sex, age, breed, and housing, were assessed for their correlation with seroreactivity. This analysis aimed to identify potential risk factors associated with rabbit exposure to *E. cuniculi*. The relationship between the seropositivity and possible associated factors, including hematologic and biochemical changes, were tested with the Chi-square (χ^2^) or Fisher’s exact test using STATA version 14.2, with *p* ≤ 0.05 considered statistically significant.

## 3. Results

**Figure 1:**
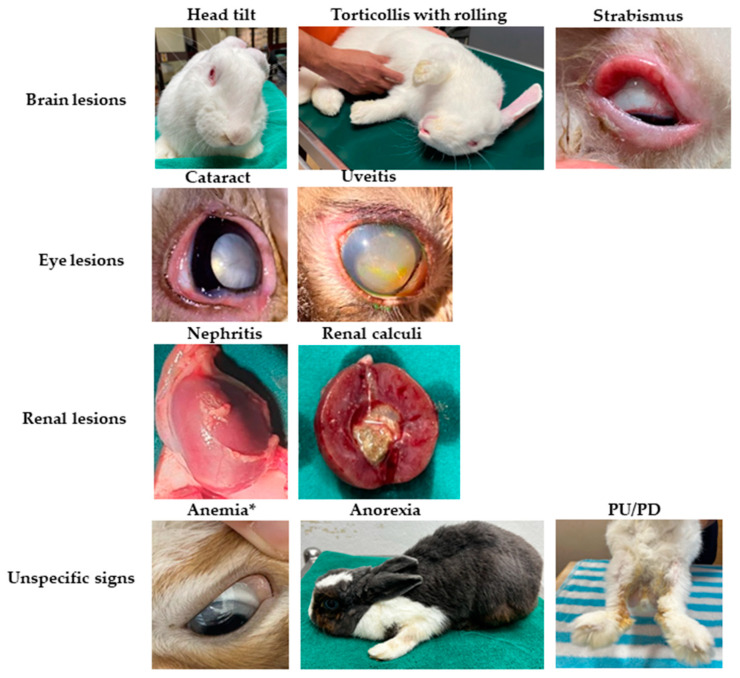
Clinical signs of rabbits with active infection with *E. cuniculi* visiting the exotic clinics. Necropsies were performed on rabbits that died during this study to examine kidney lesions and other signs of *E. cuniculi* infection. * Rabbits with anemia have a higher chance of being infected with *E. cuniculi*.

**Figure 2:**
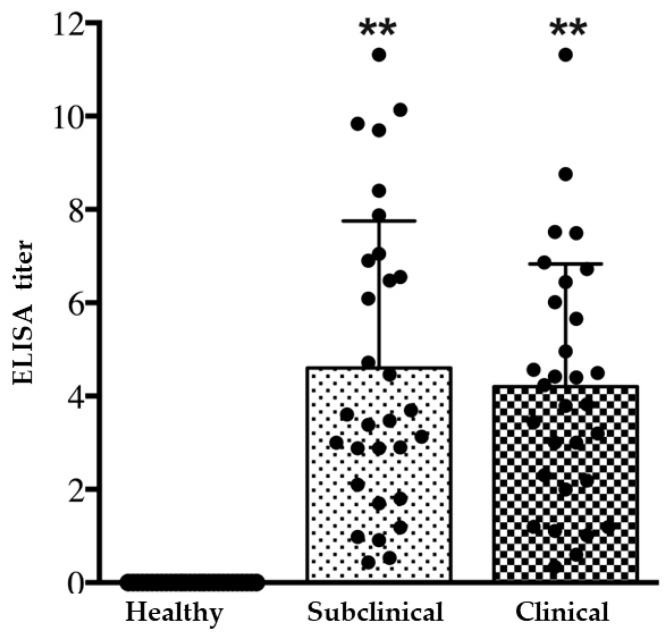
Serological titer distribution in healthy rabbits, subclinical infection, and rabbits actively infected with *E. cuniculi.* ** *p* < 0.01 vs. healthy group.

The rabbits enrolled in the present study were categorized into healthy, subclinical, and clinical groups based on their anti-*E. cuniculi* antibody status and results of the physical examination, as detailed in Table 1. The average ages of the control (37 ± 40 months) and subclinical groups (38 ± 34 months) were significantly lower than that of the clinical group (63 ± 38 months, *p* < 0.01). Despite age variations, there were no significant differences observed in clinical parameters, including hematological measures (PCV, WBC, PLT), blood chemistry (BUN, CREA, AST, ALP), and protein levels, as detailed in Table 2. Clinical signs observed in rabbits with active *E. cuniculi* infection are depicted in Table 1 and Figure 1. Rabbits exhibiting ongoing *E. cuniculi* infection typically displayed neurological signs (80.0%), including head tilt, circling, nystagmus, and strabismus. A subset of infected rabbits displayed renal lesions (10.0%) and cataracts (10.0%) as additional pathological features associated with active *E. cuniculi* infection. The serum titers in rabbits with subclinical and active infection were significantly elevated compared to the healthy control group (Figure 2). Both groups, subclinical and clinical, displayed a range of titers for anti-*E. cuniculi* antibodies. A significant difference in antibody titer was not observed between subclinical and clinical rabbits (*p* = 0.595).

The potential association between the infection status and the origin of rabbits is shown in Table 3. Among the healthy group, 60.0% of animals were obtained from pet stores, 20.0% were obtained from pet rabbit farms, and 20.0% were born in-house. In the subclinical group, 46.7% were sourced from pet stores, 46.7% were sourced from pet rabbit farms, and 6.7% were born in-house. Similarly, in the clinical group, 63.3% were sourced from pet stores, 26.7% were sourced from pet rabbit farms, and 10.0% were born in-house. There was no association between the sources of rabbits and subclinical and clinical *E. cuniculi* infection (*p* = 0.159).

The distribution of *E. cuniculi* infection was evaluated across different provinces (Table 4). Among healthy rabbits, Bangkok had the highest proportion (56.7%), followed by Saraburi (20.0%), Pathum Thani (13.3%), and Nonthaburi (6.7%), respectively. In the subclinical group, Bangkok exhibited the highest prevalence (66.7%), followed by Nonthaburi (6.7%), and Samut Prakan (10.0%), respectively. In the clinical group, Bangkok had the highest prevalence (73.3%), along with the other two provinces, Nonthaburi (23.3%), and Saraburi (3.3%), respectively.

The association between *E. cuniculi* seroprevalence and health conditions including anemia, leukocytosis, and thrombocytopenia are shown in Table 5. Anemia was not observed in the healthy and subclinical groups; however, 20.0% of animals in the clinical group exhibited anemia. *E. cuniculi* infection in the group with clinical signs was significantly linked to anemia (*p* = 0.026). No leukocytosis was detected in the healthy and subclinical groups, while the clinical group had a leukocytosis prevalence of 6.7%. A statistically significant link between *E. cuniculi* and leukocytosis (*p* = 0.613) or thrombocytopenia (*p* = 1.000) was not observed.

Analysis of the association between *E. cuniculi* and serum biochemistry parameters, including BUN, CREA, AST, ALP, and glucose levels, is shown in Table 6. No statistically significant association between *E. cuniculi* and elevated BUN (*p* = 0.104), CREA (*p* = 0.104), AST (*p* = 0.671), or ALP levels (*p* = 1.000) was determined among the three groups. Hyperglycemia was found in 36.7% of healthy rabbits, 30.0% of subclinical rabbits, and 23.3% of clinical rabbits. However, no statistically significant association between *E. cuniculi* and hyperglycemia was observed among the groups (Table 6).

## 4. Discussion

**Figure 3:**
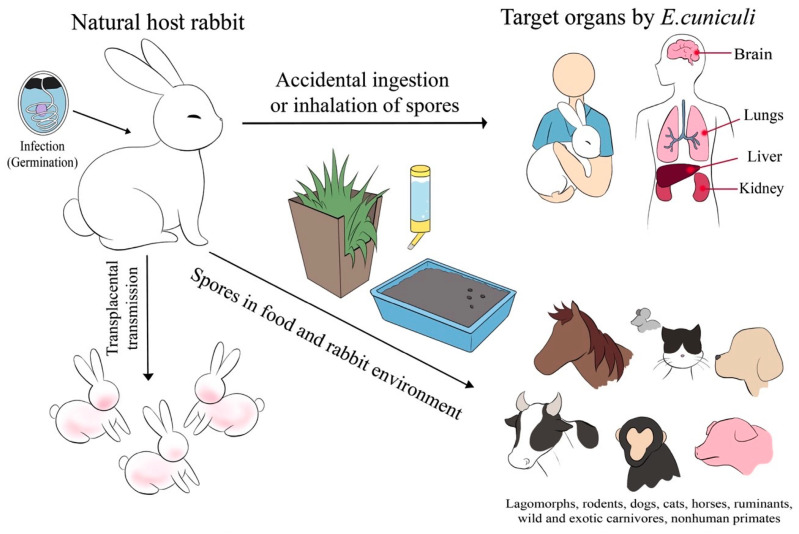
*E. cuniculi* spreads in rabbits through the following two main routes: horizontal infection through ingestion of contaminated food or water and vertical transmission from mother to offspring during gestation.

The identification of encephalitozoonosis in rabbits located in different provinces of Bangkok offers crucial insights for researchers, health professionals, veterinarians, and policymakers. *E. cuniculi* primarily disseminates through the following two primary mechanisms: horizontal transmission, wherein rabbits acquire the parasite through the ingestion of contaminated food or water, and vertical transmission, in which infected mothers transfer the pathogen to their offspring during gestation (Figure 3). These pathways demonstrate the importance of implementing stringent hygiene protocols and preventive measures to mitigate the propagation of encephalitozoonosis among rabbit populations. By controlling these pathways, the risk of transmission to humans is significantly reduced, highlighting the importance of managing *E. cuniculi* in rabbit populations to minimize potential human infection. Moreover, recommended control strategies encompass the consistent administration of antiparasitic agents, appropriate disposal methods for rabbit excreta, and the application of effective disinfectants, such as 1% sodium hypochlorite or 70% ethanol, in areas contaminated with rabbit feces [16]. Therefore, increasing public awareness and education about this disease, particularly regarding *E. cuniculi* infection in humans and animals, is essential.

Previous research has indicated that the worldwide distribution of *E. cuniculi* in pet rabbits varies from 17.9 to 97.3% in Europe, Africa, America, and Australia [17,18,19,20]. Studies in Asia have revealed a seroprevalence of *E. cuniculi* between 16.2 and 70.4% (Table 7). However, the connection between this seroprevalence and aberrant hematologic and biochemical parameters has not been thoroughly examined. The present study provides valuable insights into the relationship among *E. cuniculi* infection stages, associated clinical signs, serological titers, and hematologic and biochemical parameters. In addition, the potential influence of geographic locations in Thailand on the prevalence of *E. cuniculi* infection was investigated. The categorization of rabbits into healthy, subclinical, and clinical groups based on anti-*E. cuniculi* antibody status allowed for a comprehensive analysis of the impact of the infection.

The present findings revealed that animals in the clinical group were significantly older. This result is consistent with studies conducted in Italy and Taiwan that showed that adult rabbits, aged older than 4 months, exhibited significantly higher seropositivity for *E. cuniculi* compared to young rabbits [13,25]. It appears that *E. cuniculi* infection may become more prevalent or severe as rabbits age, perhaps due to the weakening of the immune system. *E. cuniculi* is known to proliferate rapidly in immunocompromised rabbits, resulting in clinical disease [26]. However, findings from studies conducted in Korea [24], the United Kingdom [18], and Slovenia [27] found that the age of pet rabbits was not associated with a significant increase in *E. cuniculi* infection. The reasons for these inconsistent findings are unclear, but differences in environmental factors or animal care may contribute. The diversity in our samples regarding breed, age, time of sample collection, housing, and feeding systems reflects the typical conditions under which pet rabbits are kept in these regions. While such diversity could introduce variability, it also provides a realistic representation of the conditions under which *E. cuniculi* infection occurs. We accounted for these variables in our statistical analyses, and no significant differences were observed in clinical parameters across the groups, suggesting that the impact of these variables was minimal in our study.

Encephalitozoonosis produces a wide range of clinical signs. In this study, neurological signs were the predominant clinical manifestations in *E. cuniculi*-infected rabbits. Our findings are consistent with previous reports demonstrating clinical signs of neurological disease, such as head tilt, nystagmus, strabismus, and circling [20,25,27,28,29]. *E. cuniculi* infection most often causes issues with the central nervous system, secondary to inflammation in the brain [1]. Vestibular signs of CNS lesions are typically the most common manifestation seen in encephalitozoonosis and are a useful indicator of the disease in rabbits [1]. In contrast, clinical signs of renal insufficiency are difficult to detect with azotemia and weight loss being the primary indicators [2]. Uveitis, secondary glaucoma, and cataracts can also be observed in rabbits suffering from encephalitozoonosis but these clinical signs could be attributed to other diseases [2].

Infected rabbits with *E. cuniculi* often exhibit anemia, with hematocrit levels dropping below 33%, but the precise cause of this anemia remains unclear [30]. In the present study, an association between *E. cuniculi* infection and anemia was also observed in pet rabbits. Anemia was the only relevant hematological alteration detected, present in six animals, representing approximately 20% of the clinical group and 6% of the total samples. Although anemia was noted in the infected group, it may not be solely due to *E. cuniculi* infection. *E. cuniculi* is a major cause of renal failure in rabbits, leading to granulomatous and fibrotic lesions that might impair erythropoietin production and contribute to anemia. Inflammation, a primary cause of anemia in rabbits [30,31], is connected to *E. cuniculi* infection [32] particularly in cases involving meningoencephalitis, nephritis, and the typical inflammatory lesions [33]. However, the anemia observed in our study could also result from other conditions, such as liver lobe torsion, venous endometrial aneurysms, or dental disease, which are known to cause anemia in rabbits [30,31]. Further research into the relationship among immune response, inflammatory processes, and hematological parameters, including anemia, in *E. cuniculi* clinical rabbits may aid in a better understanding of the disease process. Our analysis indicated no significant associations between *E. cuniculi* seroprevalence and serum biochemistry parameters, including BUN, CREA, AST, ALP, and glucose. However, a previous report indicated that *E. cuniculi*-infected rabbits displayed signs of renal disease and exhibited increased heterophil counts, low hematocrit changes, and changes in biochemistry renal values [2]. Elevated urea concentrations and ALP in seropositive animals and decreased phosphorus and potassium levels in *E. cuniculi*-infected rabbits have also been observed in animals with signs of renal disease [31]. In addition, in a separate study, values for BUN, CREA, ALP, cholesterol, phosphorus, and glucose were significantly higher in seropositive rabbits compared to seronegative rabbits [34]. While hematological parameters may change in advanced *E. cuniculi* infection, serum biochemistry alone is insufficient for identifying subclinical cases. The small sample size of 30 rabbits per group limits the statistical power to link anemia with renal function markers. Future research with larger sample sizes is needed to more thoroughly investigate the connections among *E. cuniculi* infection, renal disease, and anemia. Moreover, there was no statistically significant association between *E. cuniculi* seroprevalence and hyperglycemia across the groups in this study. While some rabbits exhibited hyperglycemia, this condition is likely attributable to other factors unrelated to *E. cuniculi* infection. Hyperglycemia in rabbits can be triggered by stress responses and other systemic effects, such as food intake, GI tract diseases, systemic infection, hypovolemic shock, and hyperthermia, [31,35]. Thus, it is important to recognize that the hyperglycemia observed in these rabbits may stem from these broader physiological or environmental factors rather than from *E. cuniculi* infection itself.

There is a need for more sensitive markers of infection-related changes in rabbits that may be helpful for the early detection of *E. cuniculi* infection. An important test to confirm *E. cuniculi* infection is the detection of anti-*E. cuniculi* antibodies. However, the observed elevation in anti-*E. cuniculi* antibody titers in both subclinical and clinical groups suggest that serological tests alone may not be suitable for detecting *E. cuniculi* infection due to the potential for false positives or detection of subclinical cases. An antibody titer test measures the level of free antibodies in the blood but cannot detect antibodies already attached to antigens. In severe infections, the titer may be low because many antibodies are bound to antigens, leaving fewer free antibodies. Conversely, a high titer could result from leftover antibodies from past infections or ineffective antigen–antibody complex formation. While antibody titers indicate exposure to a pathogen, they do not always directly correspond to the severity of clinical signs. Future studies examining other diagnostic tools for detecting active *E. cuniculi* infection are needed, as they could provide more accurate indications of the infection status. Furthermore, our study only included 30 infected rabbits with clinical signs, limiting our ability to comprehensively analyze potential seasonal effects. A larger sample size would be required to draw meaningful conclusions regarding seasonal patterns.

There are several limitations in the present study. This cross-sectional observational study did not utilize questionnaires to establish a causal relationship between *E. cuniculi* antibodies and clinical signs in rabbits. Additionally, there was a lack of investigation into the clinical outcomes of rabbits with a positive titer for *E. cuniculi*, as host antibodies may not necessarily affect intracellular parasitic fungi. Furthermore, the transmission of spores from rabbits, both horizontally and vertically, remains poorly defined and necessitates additional research, particularly regarding rabbit litter and environments. In addition, the current clinical biochemistry methodology exhibits limitations in sensitivity for assessing the infected status of rabbits. ELISA interpretation and potential cross-reactivity with other organisms, especially in animals naturally infected with different pathogens, could also impact the results. Variations in rabbit breeds, ages, housing, and feeding systems may further affect the findings. It is crucial to highlight that serology alone is insufficient for distinguishing different phases of infection. Given these limitations, PCR should be developed and included in future studies as a diagnostic tool for detecting active *E. cuniculi* infection, as it could provide more accurate indications of the infection status.

## 5. Conclusions

This investigation provides insights into the clinical and serological features of *E. cuniculi* infection in rabbits. The infected rabbits were mature adults, displayed neurological clinical signs, and had elevated serum titers. Anemia was significantly linked to *E. cuniculi* infection, while no significant associations were found with leukocytosis, thrombocytopenia, or various serum biochemistry parameters. Moreover, *E. cuniculi* infection did not impact serum biochemistry parameters. The source distribution and geographical analysis did not show associations with *E. cuniculi* infective stages. Pet rabbits infected with *E. cuniculi* pose a potential zoonotic risk for humans. Public health regulations should be implemented to prevent the spread of encephalitozoonosis among children and immunocompromised patients. Therefore, it is concerning that *E. cuniculi* might be transmitted from rabbits to other animals and humans involved in AAIs. Additional research is required to develop standardized health protocols to ensure comprehensive protection for both human and animal health. Serological testing alone cannot differentiate between the phases of infection, highlighting the importance of PCR for identifying active infections. Further research is required to discover novel biomarkers for the early detection of subclinical *E. cuniculi* infection and to develop improved therapeutic regimens to enhance diagnosis and treatment outcomes in rabbits.

## Figures and Tables

**Table 1:** Characteristics of the rabbit patients categorized into groups.

Parameters	Healthy	Subclinical	Clinical
*N*	30	30	30
Sex			
Male	11 (36.7%)	15 (50.0%)	13 (43.3%)
Female	19 (63.3%)	15 (50.0%)	17 (56.7%)
Age (months)	37 ± 40	38 ± 34	63 ± 38 **
Breed			
Mixed	19 (63.3%)	12 (40.0%)	12 (40.0%)
Holland Lop	8 (26.7%)	13 (43.3%)	9 (30.0%)
Netherland Dwarf	2 (6.7%)	5 (16.7%)	7 (23.3%)
Flemish Giant	1 (3.3%)	0 (0.0%)	1 (3.3%)
Rex	0 (0.0%)	0 (0.0%)	1 (3.3%)
Health status			
Normal	30 (100.0%)	30 (100.0%)	0 (0.0%)
Neurological signs	0 (0.0%)	0 (0.0%)	24 (80.0%)
Renal lesions	0 (0.0%)	0 (0.0%)	3 (10.0%)
Eye lesions	0 (0.0%)	0 (0.0%)	3 (10.0%)
IgG ELISA titer	0	4.6 ± 3.2 **	4.2 ± 2.6 **

Notes: All values are reported as rabbit numbers and percentages, ** *p* < 0.01 vs. healthy. Legend—*N* = number of rabbits; %: percentage.

**Table 2:** Hematological and biochemical parameters assessed in the rabbit patients categorized into 3 groups.

Parameters	Healthy	Subclinical	Clinical
*N*	30	30	30
PCV (%)	37 ± 3	38 ± 4	35 ± 5
WBC (cells/μL)	5649 ± 1301	6537 ± 2164	6919 ± 1950
PLT (×10^5^/L)	422.3 ± 136.3	420.6 ± 129.5	398.9 ± 112.6
BUN (mg/dL)	16 ± 5	18 ± 5	28 ± 17
CREA (mg/dL)	1.2 ± 0.2	1.3 ± 0.2	1.9 ± 1.7
AST (U/L)	44 ± 22	46 ± 28	53 ± 29
ALP (U/L)	59 ± 26	54 ± 32	49 ± 33
Protein (mg/dL)	6.3 ± 0.6	6.7 ± 0.7	6.8 ± 0.7

Notes: All values are reported as mean ± standard deviation. Legend—*N* = total rabbit numbers; PCV: packed cell volume; WBC: white blood cell count; PLT: platelet count; BUN: blood urea nitrogen; CREA: creatinine; AST: aspartate aminotransferase; ALP: alkaline phosphatase; %: percentage.

**Table 3:** Sources of rabbit with or without *E. cuniculi* infection.

Source of Rabbit	Healthy (*N* = 30)	Subclinical (*N* = 30)	Clinical (*N* = 30)
Pet store	18 (60.0%)	14 (46.7%)	19 (63.3%)
Pet rabbit farm	6 (20.0%)	14 (46.7%)	8 (26.7%)
Born in house	6 (20.0%)	2 (6.7%)	3 (10.0%)

Notes: All values are reported as rabbit numbers and percentage, Legend—*N* = rabbit numbers; %: percentage.

**Table 4:** Province of rabbits with or without *E. cuniculi* infection.

Province	Healthy (*N* = 30)	Subclinical (*N* = 30)	Clinical (*N* = 30)
Bangkok	17 (56.7%)	20 (66.7%)	22 (73.3%)
Nonthaburi	2 (6.7%)	2 (6.7%)	7 (23.3%)
Pathum Thani	4 (13.3%)	3 (10.0%)	0 (0.0%)
Saraburi	6 (20.0%)	0 (0.0%)	1 (3.3%)
Samut Prakan	0 (0.0%)	3 (10.0%)	0 (0.0%)
Roi-Et	1 (3.3%)	0 (0.0%)	0 (0.0%)
Rayong	0 (0.0%)	1 (3.3%)	0 (0.0%)
Ayutthaya	0 (0.0%)	1 (3.3%)	0 (0.0%)

**Table 5:** Association between *E. cuniculi* and anemia, leukocytosis, or thrombocytopenia in pet rabbits.

**Group**	**Anemia**	**Normal**	***P***
Healthy	0 (0.0%)	30 (100.0%)	-
Subclinical	0 (0.0%)	30 (100%)	1.000
Clinical	6 (20.0%)	24 (80.0%)	0.026
Total	6 (6.7%)	84 (93.3%)	0.003
**Group**	**Leukocytosis**	**Normal**	***P***
Healthy	0 (0.0%)	30 (100.0%)	-
Subclinical	0 (0.0%)	30 (100.0%)	1.000
Clinical	2 (6.7%)	28 (93.3%)	0.613
Total	2 (2.2%)	84 (97.8%)	0.326
**Group**	**Thrombocytopenia**	**Normal**	***P***
Healthy	0 (0.0%)	30 (100.0%)	-
Subclinical	1 (3.3%)	29 (96.7%)	1.000
Clinical	0 (0.0%)	30 (100.0%)	1.000
Total	1 (1.1%)	89 (98.9%)	1.000

Notes: All values are reported as rabbit numbers and percentage, Legend—%: percentage.

**Table 6:** Association between *E. cuniculi* and serum biochemistry (BUN, CREA, AST, ALP, glucose) in pet rabbits.

**Group**	**Elevated BUN**	**Normal**	***P***
Healthy	0 (0.0%)	30 (100.0%)	-
Subclinical	0 (0.0%)	30 (100.0%)	1.000
Clinical	5 (16.7%)	25 (83.3%)	0.104
Total	5 (5.6%)	85 (94.4%)	0.010
**Group**	**Elevated CREA**	**Normal**	***P***
Healthy	0 (0.0%)	30 (100.0%)	-
Subclinical	0 (0.0%)	30 (100.0%)	1.000
Clinical	5 (16.7%)	25 (83.3%)	0.104
Total	5 (5.6%)	85 (94.4%)	0.010
**Group**	**Elevated AST**	**Normal**	***P***
Healthy	1 (3.3%)	29 (96.7%)	-
Subclinical	2 (6.6%)	28 (93.3%)	1.000
Clinical	4 (13.3%)	26 (86.7%)	0.671
Total	7 (7.8%)	83 (98.9%)	0.493
**Group**	**Elevated ALP**	**Normal**	***P***
Healthy	1 (3.3%)	29 (96.7%)	-
Subclinical	1 (3.3%)	29 (96.7%)	1.000
Clinical	2 (6.6%)	28 (93.3%)	1.000
Total	4 (4.4%)	86 (95.6%)	1.000
**Group**	**Hyperglycemia**	**Normal**	***P***
Healthy	11 (36.7%)	19 (63.3%)	-
Subclinical	9 (30.0%)	21 (70.0%)	0.785
Clinical	7 (23.3%)	23 (76.7%)	0.399
Total	27 (30.0%)	63 (70.0%)	0.576

Notes: All values are reported as rabbit numbers and percentage, Legend—BUN: blood urea nitrogen; CREA: creatinine; AST: aspartate aminotransferase; ALP: alkaline phosphatase; %: percentage.

**Table 7:** Seroprevalence of *E. cuniculi* in rabbits reported previously from various Asian countries, including Thailand.

Country	Rabbit Population	Prevalence (%)	Serological Method	References
China	Pet rabbits	18.7	ELISA	[21]
		16.2	ELISA	[22]
Japan	Pet rabbits	63.5 (IgG)	ELISA	[23]
		28.5 (IgM)	ELISA	[23]
Korea	Pet rabbits	22.6	ELISA	[24]
Taiwan	Pet rabbits	67.8	ELISA	[25]
		63.2	CIA	[25]
Thailand	Meat rabbits	42.4–70.4	ELISA	[15]
	Pet rabbits	67.1	ELISA	[15]
	Pet rabbits	66.7	ELISA	Present study

## Data Availability

The datasets generated and/or analyzed during the current study are available from the corresponding author upon reasonable request.
